# Are Social Relationships an Underestimated Resource for Mental Health in Persons Experiencing Physical Disability? Observational Evidence From 22 Countries

**DOI:** 10.3389/ijph.2021.619823

**Published:** 2021-04-16

**Authors:** Christine Fekete, Hannah Tough, Mohit Arora, Nazirah Hasnan, Conran Joseph, Daiana Popa, Vegard Strom, James Middleton

**Affiliations:** ^1^ Swiss Paraplegic Research, Nottwil, Switzerland; ^2^ Department of Health Sciences and Medicine, University of Lucerne, Lucerne, Switzerland; ^3^ John Walsh Centre for Rehabilitation Research, Kolling Institute of Medical Research, Royal North Shore Hospital, St. Leonards, NSW, Australia; ^4^ Sydney Medical School - Northern, Faculty of Medicine and Health, The University of Sydney, Sydney, NSW, Australia; ^5^ Department of Rehabilitation Medicine, Faculty of Medicine, University of Malaya and UM Medical Centre, Kuala Lumpur, Malaysia; ^6^ Faculty of Medicine and Health Science, Department of Health and Rehabilitation Sciences, Physiotherapy Division, Stellenbosch University, Cape Town, South Africa; ^7^ Clinical Rehabilitation Hospital Felix-Spa Bihor County, Oradea, Romania; ^8^ Sunnaas Rehabilitation Hospital, Nesoddtangen, Norway

**Keywords:** mental health, social relationships, MHI-5, physical disabilities, spinal cord injury, insci community survey

## Abstract

**Objectives:** As advancing evidence on modifiable resources to support mental health in persons experiencing physical disabilities is of particular importance, we investigate whether structural and functional social relationships relate to mental health in people with spinal cord injury (SCI).

**Methods:** Data from 12,330 participants of the International SCI community survey (InSCI) from 22 countries were analyzed. Structural (partnership status, living situation) and functional aspects of social relationships (belongingness, relationship satisfaction, problems with social interactions) were regressed on the SF-36 mental health index (MHI-5), stratified by countries and for the total sample using multilevel models.

**Results:** Functional aspects of social relationships were consistently related to clinically relevant higher MHI-5 scores and lower risk of mental health disorders (MHI-5 >56). Structural social relationships were inconsistently associated with mental health in our sample.

**Conclusion:** This study provides evidence that functional aspects of social relationships are important resources for mental health. Interventions to establish and maintain high quality relationships should be considered in public health interventions and rehabilitation programs to reduce long-term mental health problems in persons experiencing physical disabilities.

## Introduction

Mental health has long been recognized as important public health issue [1] and poor mental health represents a major burden of disease in people experiencing physical disabilities [2]. Advancing evidence on modifiable resources to support mental health in this population is thus essential. One set of modifiable resources to strengthen mental health are social relationships [3–6], which need to be acknowledged as public health priorities, given their powerful impact on population health [7]. Although there is little conceptual consensus, a basic distinction into structural and functional aspects of social relationships is widely accepted and helpful to illustrate the diversity of constructs [8]. *Structural aspects* reflect quantitative characteristics of social relationships, such as social network size, frequency of social contacts, the living situation or partnership status, while *functional aspects* include *qualitative* features of relationships. The most frequently studied construct in the latter category is social support, which describes the functional importance of social relationships in their ability to provide instrumental, emotional or informational resources [8, 9]. Other functional aspects of relationships capture the emotional appraisal of social relationship, such as feelings of belongingness or relationship satisfaction. Earlier research suggests that functional aspects are more important for mental health than structural aspects as the psychosocial mechanisms through which social relationships affect mental health mainly depend on their functionality and less so on the pure size or structure of networks. Poor functionality or quality of social relationships may detrimentally affect psychological aspects (e.g., emotional regulation, self-esteem, coping) [10]; and provoke harmful physiological reactions, such as the activation of the hypothalamic-pituitary-adrenal axis, which leads to increased susceptibility for allostatic load, adverse cardiovascular responses, and inflammation [11–13], impeding mental health in the long-term.

Although evidence for the beneficial effects of social relationships on mental health from general population samples in higher income settings is far advanced and fairly consistent [3–6], findings from populations with physical disabilities are less conclusive. For structural relationships, evidence is mainly available for the size of social networks, and results of their association with general mental health, depression and anxiety are mixed [14–17]. A recent systematic review on findings from populations with physical disabilities showed that only 59% of included studies confirmed a negative association between various aspects of social support and depression, and weak associations between social support and anxiety [16]. This inconclusiveness might partly be explained by the potentially adverse effects of receiving social support in disability as unwanted or unnecessary instrumental social support may lead to reduced autonomy, self-worth and self-esteem, which can negatively affect mental health [18]. Further, it might be particularly difficult to exclude reverse causation in people with physical disabilities, as an increased receipt of social support may not reflect a resource for mental health per se, but results from an increased need of informal care due to the physical impairments. Constructs focusing on the perceived quality or emotional appraisal of social relationships might be less prone to bias in the setting of disability, however, there is a dearth of research on functional aspects of social relationships other than social support, such as relationship quality and loneliness-related constructs [16].

The present study provides the opportunity for two major contributions to current literature on social relationships and mental health in the setting of physical disability by using a large sample of people with spinal cord injury (SCI) from 22 countries. First, this study not only investigates structural aspects of social relationships (i.e., partnership status, living situation) but also functional aspects that have rarely been studied in disability research (i.e., belongingness, relationship satisfaction, problems with social interactions). Second, the use of international data allows the investigation of associations within different countries to assess whether the expected link between social relationships and mental health is observed universally. We use data from people with SCI as a model for physical disability, as this condition often has severe consequences on functioning and health and increased vulnerability to psychological morbidity [19]. Traumatic or non-traumatic injury to the spinal cord causes complete or partial loss of motor function and sensation below the lesion level [20], and results of our study might be generalizable among persons with mobility impairments and limitations in activities of daily living. In summary, this study aims to examine associations of structural and functional aspects of social relationships with mental health in people with SCI from 22 countries.

## Design

The International Spinal Cord Injury community survey (InSCI) is a cross-sectional survey performed in 22 countries between January 2017 and May 2019, covering all six World Health Organization regions. People with traumatic or non-traumatic SCI over 18 years of age who lived in the community and were able to respond to the available questionnaire language version were included. People with neuro-degenerative disorders, congenital etiologies, or Guillain Barré syndrome were excluded from the study given their different rehabilitation paths and disease progressions than persons with acquired SCI [21]. National Study Centers were responsible for recruitment and data collection. Sampling frames were developed based on local conditions and included random and convenience sampling. Random sampling based on predefined sampling frames was applied in 8 countries with access to hospital or patient organization databases. Since access to such databases was lacking, 14 countries used convenience sampling for recruiting individuals visiting health care facilities or joining patient organization events [22]. Power analysis indicated a minimum of 200 participants per country [21]. Ten countries recruited between 200 and 300 participants given that detecting potential participants was challenging because SCI is a rare condition and systematic records and relevant databases were missing or not accessible. Response rates (% of participants among total of eligible, including those who could not be contacted) for the 8 countries applying random sampling ranged from 22.8% in China to 53.6% in South Africa and cooperation rates (% of participants among total of eligible who could be contacted) ranged from 29.4% in Australia to 89.7% in South Africa. Details on response rates of random-sampling countries, recruitment outcomes and participant characteristics of all InSCI countries are reported elsewhere [22].

Response modes included paper-pencil or online questionnaires, telephone or personal interviews and countries were responsible to provide the most convenient response modes. All countries received regulatory approvals by Institutional Review Boards or Ethics Committees and compliance with national laws and conformity with the Helsinki Declaration was guaranteed. Each participant provided informed consent in accordance with national regulations. Further information on the rationale, methodology, recruitment outcomes, and basic sample characteristics of the InSCI community survey are reported elsewhere [21–23].

## Measures

### Predictors: Social Relationships

*Structural aspects of social relationships* were operationalized by partnership status and living situation. Partnership status was assessed by an item asking participants about their current marital status including the response options single; married; cohabiting or in a partnership; separated or divorced; and widowed. People who reported being married, cohabiting or living in a partnership were categorized as “having a partner” and others as “having no partner”. Information on the living situation was gathered by an item asking participants whether they lived alone, whether and how many other people lived in the same household, or whether they lived in an institution (e.g., nursing home, home for the elderly). A three-categorical variable was constructed discriminating between living alone, living with others, and living in an institution.

*Functional aspects of social relationships* were assessed with information on feelings of belongingness, satisfaction with social relationships and problems with social interactions. Belongingness was measured with an item from the General Belongingness Scale [24] asking participants whether they feel included when they are with other people. The purpose of this item is to capture a general feeling of connectedness or an overarching sense of belongingness, describing the phenomenon that the feeling of low belongingness can also occur if people are surrounded by others. The belongingness item was rated on a 5-point scale ranging from 0 (not at all) to 4 (completely). A WHOQoL-BREF item was used to measure relationship satisfaction [25]. Participants were asked about their satisfaction with their personal relationships on a 5-point scale ranging from 0 (very dissatisfied) to 4 (very satisfied). Three items from the Model Disability Survey were used to measure the extent of problems with social interactions [26]. The items were formulated as follows: "In the past 4 weeks, how much of a problem did you have with 1) …providing care or support for others; 2) …interacting with people; 3) …intimate relationships". Response options included a scale from 0 (extreme problem) to 4 (no problem) and we calculated a sum score over the 3 items (0–12), with higher scores indicating fewer problems with social interactions.

### Outcome: Mental Health

The SF-36 5-item Mental Health Index (MHI-5, version 2) was used to assess general mental health [27]. The MHI-5 showed satisfactory reliability and validity as a screening instrument for general mental health problems in individuals with SCI [28] and measures the frequency of emotional states in the past four weeks on a 5-point scale from 0 (all of the time) to 4 (none of the time). The raw score was transformed to a 0–100 scale according to established guidelines [27]. Additionally, the MHI-5 scale was dichotomized based on recommendations for mental health monitoring in Europe [29] in order to distinguish people with high risk of experiencing mental health disorders (scores ≤ 56) from those with a low risk (scores > 56).

### Potential Confounders

The approach of directed acyclic graphs (DAG) was used to inform the selection of confounders by exploring correlations between potential confounders, predictors and outcomes [30]. We selected candidate confounders based on the literature and only variables that were related to predictors and outcome variables in our data were entered as confounders into final models. Age, gender, employment status, socioeconomic position, SCI severity, time since injury, access to health care, and secondary health conditions were identified as confounders, given their association with the predictors and the outcomes. Partnership status was additionally included for models on living situation and functional aspects of social relationships.

To operationalize socioeconomic position (SEP), we built a sum score based on information about years of education (quartiles per country; 0–3), net-equivalent household income (quartiles per country; 0–3), subjective social position (low, middle, high; 0–2), and financial hardship (none, some, great; 0–2). Given that the number of people with lowest and highest scores were rare (0 points: *n* = 89; 9–10 points: *n* = 98), we pooled people scoring 0 into the lowest (combining scores 0–1) and people scoring 9 or 10 into the highest SEP group (combining scores 8–10). This resulted in an SEP score ranging from 0–8. SCI severity was grouped into four categories (incomplete paraplegia; complete paraplegia; incomplete tetraplegia; complete tetraplegia) based on information concerning level and completeness of the injury. The completeness of injury was evaluated with an item asking participants whether their injury was complete or incomplete, including respective definitions (complete being defined as “unable to feel and move any part of your body below injury level”; incomplete as “able to feel or move some part/s of your body below injury level”). Employment status was assessed with a multiple-choice question about the current employment status, whereby those indicating to work for salary with an employer or being self-employed were coded as having paid work, and those not selecting either of those categories were coded as having no paid work. Access to health care was assessed with a binary item asking participants whether they were in need of health care but did not get it during the past 12 months. Secondary health conditions were assessed with 14 items of the SCI-Secondary Conditions Scale (SCI-SCS) [31] with modified response options rated on a 5-point scale from 0 (no problem) to 4 (extreme problem). Switzerland used the original SCI-SCS scoring scheme to maintain longitudinal comparability with earlier surveys and applied a 4-point scale ranging from 0 (no/mild problem) to 3 (significant/chronic problem). The items were harmonized into 0 (no), 1 (some), or 2 (severe problem), whereby 0 in both scales was scored 0; 1 and 2 in both scales was scored 1; and 3 and 4 for the international scale and 3 for the Swiss scale was scored with 2. This 0–2 scoring scheme resulted in a 0–28 sum score.

## Statistical Analysis

Analyses were conducted using STATA version 16.0 for Windows (College Station, TX, USA). Unadjusted descriptive analysis of distributions of main variables of interest are presented pooled for the total sample and predictor and outcome variables are additionally given stratified for all countries. Association between structural and functional aspects of social relationships with mental health were investigated in the total sample as well as stratified for countries, to explore whether associations were observed universally. For analysis on the total sample, we ran multilevel regressions with a random intercept for country to account for the clustering of data within countries and to adjust for country affiliation [32]. Secondly, regression models were run separately for the 22 countries. For multilevel as well as country-specific regressions, we used models for continuous (sum score of general mental health, 0–100) and binary outcomes (high vs. low risk of mental health disorders). Coefficients and odds ratios (OR) with 95% confidence intervals (CI) and *p*-values from likelihood ratio tests were reported for all models. We present unadjusted and adjusted results, controlling adjusted models for relevant confounders described above.

Missing values were imputed by multiple imputation (MI), assuming that the missing values were not related to the variable with the missing value itself (i.e., missing completely at random). To impute different types of variables, MI by chained equations (MICE) was applied [33]. Imputation was performed on item level for all variables. Descriptive results are shown for crude data and results from regression modeling are based on imputed data. Results from full case analysis and analysis based on imputed data were compared in sensitivity analyses and no differences between the two strategies were observed (results not shown).

## Results

A description of the total sample is displayed in Table 1 and summary statistics for main variables of interest are given stratified by countries in the Supplementary Tables S1, S2. Around three-quarters of the sample were male, with mean age around 51 years and nearly one-third having paid work. Incomplete paraplegia was the most frequent diagnosis (35%) and complete tetraplegia the least frequent diagnosis (10%). The SCI was caused by trauma in 80% of people and mean time since injury was around 13 years. Eighteen percent of the sample reported having not received medical treatment over the past 12 months despite needing it. On average, the problem extent of secondary health conditions was rated 10 on a 0–28 scale. Fifty-nine percent of the sample lived in a partnership, 78% of people lived with others and roughly 3% lived in an institution. Mean scores on the 0–4 belongingness and relationship satisfaction scales were 2.8 and 2.7, respectively. Around one-third of people reported feeling completely included when being with others and two-thirds of people were satisfied or very satisfied with their social relationships. On a 0–12 scale, people indicated an average score of 7.7 for problems with social interactions, with higher scores indicating fewer problems with interactions. The mean score on the 0–100 MHI-5 scale was 66.2 and one-third scored below 57, indicating a high risk for mental health disorders.

**TABLE 1:** Basic characteristics of the 12,330 participants of the International Spinal Cord Injury Community Survey (InSCI, 2017–2019).

Categorical variables [% of missing values in total sample]	N (%)	Mean (SD); median (IQR)
Sociodemographic characteristics		
Gender [0.3]		
Male	8,974 (73.0)	
Female	3,323 (27.0)	
Age at time of survey in years [0.6]		51.2 (15.2); 52 (40–62)
Employment status [0]		
Paid work	3,794 (30.8)	
No paid work	8,536 (69.2)	
Socioeconomic position, range 0–8 [19.9]		4.5 (1.9); 4 (3–6)
Lesion characteristics		
Severity of SCI [4.0]		
Incomplete paraplegia	4,071 (34.4)	
Complete paraplegia	3,335 (28.2)	
Incomplete tetraplegia	3,233 (27.3)	
Complete tetraplegia	1,196 (10.1)	
Etiology [1.6]		
Traumatic	9,797 (80.7)	
Non-traumatic	2,337 (19.3)	
Time since injury in years [2.5]		13.0 (11.8); 9 (4–19)
Health and health care indicators		
No health care although needed [3.1]	2,168 (18.2)	
Secondary health conditions, range 0–28 [14.3]		10.4 (5.7); 10 (6–14)
Social relationships		
Partnership status [0.7]		
Having a partner	7,236 (59.1)	
Having no partner	5,003 (40.9)	
Living situation [1.5]		
Living alone	2,230 (18.4)	
Living with others	9,506 (78.3)	
Living in an institution	405 (3.3)	
Feelings of belongingness, range 0–4 [2.8]		2.8 (1.1); 3 (2–4)
0 = not at all	498 (4.2)	
1	1,057 (8.8)	
2	2,640 (22.0)	
3	3,778 (31.5)	
4 = completely	4,011 (33.5)	
Relationship satisfaction, range 0–4 [2.5]		2.7 (1.0); 3 (2–3)
0 = very dissatisfied	350 (2.9)	
Dissatisfied	989 (8.2)	
Neither nor	2,801 (23.3)	
Satisfied	5,733 (47.7)	
4 = very satisfied	2,150 (17.9)	
Social interactions, 0 = no problems, 12 = extreme problems [3.1]		7.7 (3.4); 8 (5–11)
Mental health [3.1]		
General mental health SF-36 MHI-5, range 0–100		66.2 (20.6); 68 (50–82)
Risk of mental health disorder		
Low risk (MHI-5 >56)	7,997 (67.4)	
High risk (MHI-5 ≤56)	3,865 (32.6)	

Abbreviations: IQR, inter-quartile range; MHI-5: SF-36 5-item mental health index, higher scores indicating better mental health; SCI: spinal cord injury; SD: standard deviation.

### Total Sample Analysis

Results for the total sample are shown in Table 2. People having a partner reported marginally higher mental health scores and lower likelihood for mental health disorders. People living with others showed slightly higher mental health scores than those living alone, while people living in institutions reported lower mental health scores compared to those living alone. However, the differences between the groups with different living situations were not observed when considering the dichotomous outcome variable on risk of mental health disorders. Functional aspects of social relationships were consistently related to general mental health and the odds of reporting a low risk of mental health disorders. With each increase on the belongingness or relationship satisfaction scale, the score on the general mental health scale and the likelihood for not having a mental health disorder markedly increased. Each unit of increase on the social interactions scale was associated with 1.98 points rise on the general mental health scale and a 1.22 higher likelihood to report a low risk of a mental health disorders.

**TABLE 2:** Social relationships and mental health pooled for the 22 countries participating in the International Spinal Cord Injury Community Survey (InSCI): Results from crude and adjusted multilevel regressions, coefficients or odds ratios and 95% confidence intervals (InSCI, 2017–2019).

	General mental health(MHI-5 scores 0–100)		Low risk of mental health disorder(MHI-5 scores >56)	
AdjustmentEffect sizes	Model 1	Model 2	Model 1	Model 2
	Coeff (95% CI)	Coeff (95% CI)	OR (95% CI)	OR (95% CI)
**Structural aspects of social relationships**				
Partnership status				
No partner	References	References	References	References
Having a partner	2.15 (1.40–2.90)	1.25 (0.55–1.95)	1.17 (1.08–1.27)	1.11 (1.01–1.22)
*p*-value	<0.001	<0.001	<0.001	0.027
Living situation				
Living alone	References	References	References	References
Living with others	1.86 (0.89–2.83)	0.92 (−0.11–1.96)	1.10 (0.99–1.22)	1.02 (0.89–1.17)
Living in an institution	−3.68 (5.95–−1.40)	−1.72 (−3.76–0.32)	0.67 (0.53–0.85)	0.78 (0.61–1.00)
*p*-value	<0.001	0.013	<0.001	0.084
**Functional aspects of social relationships**				
Belongingness				
0 = not at all	References	References	References	References
1	2.67 (0.62–4.73)	2.64 (0.75–4.54)	1.04 (0.83–1.31)	1.07 (0.83–1.37)
2	9.75 (7.90–11.61)	7.65 (5.95–9.34)	1.94 (1.57–2.39)	1.74 (1.39–2.18)
3	18.52 (16.73–20.31)	13.92 (12.26–15.58)	4.76 (3.87–5.86)	3.58 (2.86–4.48)
4 = completely	24.55 (22.73–26.37)	18.36 (16.68–20.03)	7.67 (6.21–9.48)	5.33 (4.24–6.70)
*p*-value	<0.001	<0.001	<0.001	<0.001
Relationship satisfaction				
Very low	References	References	References	References
Low	5.11 (2.87–7.36)	3.38 (1.33–5.45)	1.26 (0.96–1.66)	1.09 (0.82–1.47)
Medium	15.15 (13.10–17.20)	11.35 (9.46–13.23)	2.81 (2.19–3.61)	2.22 (1.70–2.91)
High	25.78 (23.79–27.77)	19.58 (17.73–21.42)	8.02 (6.27–10.24)	5.58 (4.28–7.28)
Very high	32.34 (30.26–34.43)	24.73 (22.78–26.67)	14.19 (10.82–18.61)	9.17 (6.85–12.30)
*p-value*	<0.001	<0.001	<0.001	<0.001
Social interactions				
0 = extreme problems to 12 = no problems	2.83 (2.73–2.94)	1.98 (1.86–2.09)	1.31 (1.29–1.33)	1.22 (1.20–1.24)
*p*-value	<0.001	<0.001	<0.001	<0.001

Abbreviations: CI: Confidence intervals; Coeff: Coefficients; MHI-5: SF-36 5-item Mental Health Index; OR: Odds ratios. Results based on imputed data (*n* = 12,330).

Model 1: unadjusted; Model 2 adjusted for age, gender, partnership status, employment status, socioeconomic position, lesion characteristics, unavailability of health services and secondary health conditions. *p-values* from likelihood ratio tests.

Reading examples.

*Relationship satisfaction, Model 2, 'General mental health'*: People with low relationship quality report 3.31 points more on the 0–100 mental health scale as compared to those with very low relationship satisfaction; people with very high relationship quality report 24.6 points more on the 0–100 mental health scale as compared to those with very low relationship satisfaction.

*Relationship satisfaction, Model 2, 'Low risk of mental health disorder*: The likelihood to report a low risk of mental health disorder increases 1.09-times for persons with low relationship satisfaction, 2.22-times for those with medium relationship quality, 5.58-times for those with high relationship satisfaction and 9.17-times for those with very high relationship satisfaction as compared to persons with very low relationship satisfaction.

### Country-specific Analysis

Results from country-specific analysis for the 0–100 general mental health score and for the dichotomized score (high vs. low risk of mental health disorders) are displayed in Tables 3 and 4, respectively. The association of partnership status and living situation with mental health was inconsistent across countries, however, results for functional aspects of social relationships and mental health confirm findings from the total sample in a large majority of countries. We observe that increased feelings of belongingness, higher relationship satisfaction and fewer problems with social interactions were related to higher scores on the general mental health scale and much lower risk of mental health disorders across most countries.

**TABLE 3:** Social relationships and general mental health in the 22 countries participating in the International Spinal Cord Injury Community Survey (InSCI): adjusted **coefficients** and 95% confidence intervals for general mental health by social relationships, stratified by country (InSCI 2017–2019).

	General mental health (MHI-5 scores 0–100)																					
	Australia	Brazil	China	France	Germany	Greece	Indonesia	Italy	Japan	Lithuania	Malaysia	Morocco	Netherlands	Norway	Poland	Romania	South Africa	South Korea	Spain	Switzerland	Thailand	United States
	Coeff 95% CI	Coeff 95% CI	Coeff 95% CI	Coeff 95% CI	Coeff 95% CI	Coeff 95% CI	Coeff 95% CI	Coeff 95% CI	Coeff 95% CI	Coeff 95% CI	Coeff 95% CI	Coeff 95% CI	Coeff 95% CI	Coeff 95% CI	Coeff 95% CI	Coeff 95% CI	Coeff 95% CI	Coeff 95% CI	Coeff 95% CI	Coeff 95% CI	Coeff 95% CI	Coeff 95% CI
Partnership status																						
No partner	Ref	Ref	Ref	Ref	Ref	Ref	Ref	Ref	Ref	Ref	Ref	Ref	Ref	Ref	Ref	Ref	Ref	Ref	Ref	Ref	Ref	Ref
Having a partner	**2.12**	**2.66**	**-0.20**	**1.38**	**2.62**	**6.77**	**0.89**	**3.06**	**4.62**	**1.20**	**2.92**	**-6.89**	**3.54**	**0.77**	**0.09**	**0.51**	**4.74**	**5.00**	**-1.52**	**-0.40**	**2.87**	**-1.84**
	0.07–4.17	−3.97–9.30	−2.96–2.55	−2.08–4.84	0.64–4.61	0.45–13.09	−5.00–6.78	−1.29–7.41	−0.01–9.25	−3.58–5.98	−1.39–7.24	−11.91–−1.88	−1.39–8.47	−2.02–3.56	−2.30–2.48	−4.58–5.60	−1.83–11.31	2.11–7.98	−5.86–2.82	−2.30–1.50	−1.52–7.27	−7.07–3.40
*p*-value	0.043	0.429	0.884	0.433	0.010	0.036	0.767	0.167	0.051	0.622	0.184	0.007	0.159	0.588	0.941	0.843	0.156	0.001	0.492	0.680	0.199	0.489
Living situation																						
Living alone	Ref	Ref	Ref	Ref	Ref	Ref	Ref	Ref	Ref	Ref	Ref	Ref	Ref	Ref	Ref	Ref	Ref	Ref	Ref	Ref	Ref	Ref
Living with others	**−0.88**	**−4.55**	**3.64**	**−1.46**	**3.64**	**−3.49**	3.68	**−3.19**	**2.61**	**9.85**	**2.02**	**9.34**	**2.85**	**0.38**	**1.36**	**−6.08**	**−2.24**	**0.36**	**−0.87**	**1.74**	**−0.49**	−2.94
	−3.72–1.96	−17.33–8.23	−0.70–7.99	−6.95–4.03	0.67–6.62	−11.26–4.28	−7.44–14.81	−9.34–2.95	−4.15–9.38	2.45–17.25	−6.37–10.42	−1.32–20.01	−6.46–12.15	−3.96–4.71	−2.48–5.20	−14.09–1.93	−13.21–8.73	−3.09–3.82	−7.52–5.78	−1.19–4.67	−11.17–10.20	−9.94–4.06
Living in an	**−2.52**	N/A	**−8.19**	**0.13**	**−0.44**	**−24.44**	**5.20**	**−10.52**	**−8.27**	N/A	**−8.33**	**20.82**	**8.85**	**−12.78**	**3.23**	**31.86**	**−11.06**	**−1.00**	**0.58**	**2.18**	**2.18**	**−0.49**
institution	−8.09–3.04		**−**15.36–−1.01	−13.81–14.07	**−**6.22–5.34	−66.20–17.31	−7.31–17.72	−26.39–5.35	**−**25.17–8.63		−20.36–3.69	−5.30–46.94	**−**7.49–25.19	**−**21.78–−3.77	**−**3.38–9.84	**−**2.73–66.44	−23.25–1.13	**−**13.91–11.91	**−**14.89–16.05	**−**3.31–7.68	−10.31–14.67	**−**32.48–31.49
*p*-value	0.632	0.484	<0.001	0.861	0.051	0.394	0.719	0.313	0.341	0.009	0.076	0.136	0.518	0.013	0.605	0.038	0.054	0.961	0.956	0.442	0.812	0.704
Belongingness																						
0 = not at all	Ref	Ref	Ref	Ref	Ref	Ref	Ref	Ref	Ref	Ref	Ref	Ref	Ref	Ref	Ref	Ref	Ref	Ref	Ref	Ref	Ref	Ref
1	**3.30**	**10.74**	**7.63**	**1.42**	**2.78**	**−8.33**	**1.48**	**2.23**	**6.74**	**−10.47**	**−3.17**	**14.69**	**0.91**	**5.11**	**−1.58**	**16.81**	**−0.81**	**4.99**	**−19.54**	**−2.97**	**4.98**	**−0.00**
	**−**2.00–8.60	**−**3.69–25.17	3.30–11.97	**−**10.71–13.55	**−**3.91–9.45	**−**27.97–11.31	**−**8.76–11.72	**−**8.70–13.15	**−**1.87–15.35	**−**49.25–28.31	**−**12.24–5.89	3.45–25.93	**−**12.99–14.80	−13.11–23.33	**−**7.99–4.83	0.91–32.71	**−**18.48–16.86	**−**0.76–10.75	**−**32.99–−6.08	**−**10.36–4.41	**−**8.64–18.59	**−**20.59–20.58
2	**6.25**	**2.74**	**16.20**	**8.52**	**5.56**	**4.38**	**0.12**	**8.94**	**14.19**	**14.73**	**1.90**	**18.83**	**−4.17**	**6.33**	**6.88**	**5.46**	**−0.56**	**10.09**	**−5.01**	**0.50**	**12.80**	**1.27**
	1.42–11.07	**−**8.73–14.20	12.20–20.20	**−**2.89–19.94	**−**0.43–11.55	**−**13.60–22.35	**−**9.15–9.39	−1.71–19.60	6.48–21.90	**−**6.73–36.18	**−**6.03–9.83	8.62–29.05	**−**16.97–8.63	**−**11.22–23.88	1.24–12.51	**−**8.49–19.41	**−**14.52–13.40	4.94–15.23	**−**15.83–5.80	−6.02–7.03	−0.04–25.64	**−**18.47–21.02
3	**13.45**	**23.75**	**23.21**	**14.12**	**9.85**	**11.09**	**2.89**	**11.57**	**24.44**	**22.46**	**6.23**	**22.71**	**1.60**	**8.89**	**12.41**	**12.36**	**9.91**	**19.79**	**1.40**	**7.39**	**15.83**	**4.97**
	8.57–18.33	12.31–35.19	19.12–27.29	2.82–25.42	4.02–15.68	**−**6.09–28.27	**−**6.19–11.98	1.32–21.82	16.41–32.47	1.57–43.35	**−**1.33–13.78	12.45–32.96	**−**10.92–14.13	**−**8.74–26.52	7.07–17.74	−1.73–26.45	**−**3.64–23.45	14.52–25.07	**−**8.66–11.46	1.18–13.59	2.99–28.67	**−**14.47–24.41
4 = completely	**18.79**	**22.44**	**25.42**	**17.30**	**14.39**	**18.93**	**6.42**	**17.37**	**24.52**	**27.48**	**10.50**	**29.94**	**6.71**	**15.16**	**15.96**	**16.92**	**9.16**	**21.75**	**5.94**	**11.95**	**16.70**	**13.23**
	13.95–23.63	12.61–32.27	20.99–29.84	6.12–28.48	8.54–20.24	1.86–36.00	**−**3.24–16.09	6.77–27.97	16.32–32.72	6.58–48.37	2.71–18.29	20.50–39.39	**−**5.94–19.37	−2.29–32.62	10.65–21.27	3.09–30.75	**−**4.30–22.61	15.91–27.58	**−**3.67–15.56	5.75–18.15	3.62–29.77	**−**6.34–32.81
*p*-value	<0.001	<0.001	<0.001	<0.001	<0.001	<0.001	0.527	<0.001	<0.001	<0.001	<0.001	<0.001	0.020	<0.001	<0.001	0.003	0.025	<0.001	<0.001	<0.001	002	<0.001
Relationship satisfaction																						
Very low	Ref	Ref	Ref	Ref	Ref	Ref	Ref	Ref	Ref	Ref	Ref	Ref	Ref	Ref	Ref	Ref	Ref	Ref	Ref	Ref	Ref	Ref
Low	**3.52**	**−10.25**	**12.07**	**6.63**	**7.09**	**12.38**	**−22.61**	**6.80**	**5.77**	**23.12**	**−12.26**	**7.68**	**−4.18**	**7.89**	**1.44**	**−2.78**	**−0.27**	**5.70**	**6.63**	**−8.87**	**16.09**	**8.65**
	**−**1.13–8.17	−32.44–11.94	2.42–21.73	−5.01–18.27	1.35–12.83	**−**6.78–31.53	**−**39.52–−5.70	**−**4.17–17.77	**−**5.39–16.93	9.29–36.95	**−**27.78–3.26	−6.22–21.59	**−**22.53–14.18	**−**12.33–28.11	**−**7.33–10.21	**−**19.59–14.04	**−**17.84–17.30	0.13–11.27	**−**6.61–19.87	**−**15.92–−1.82	−18.48–50.66	**−**9.43–26.73
Medium	**11.60**	**−9.82**	**20.45**	**14.28**	**16.04**	**16.27**	**−13.35**	**13.66**	**18.18**	**17.99**	**−6.39**	**15.39**	**5.49**	**18.73**	**5.74**	**1.76**	**2.81**	**15.37**	**8.81**	**2.75**	**33.81**	**14.43**
	7.30–15.90	**−**29.51–9.87	11.31–29.59	3.71–24.84	10.61–21.48	**−**2.41–34.94	−28.25–12.30	2.76–24.57	7.51–28.85	6.41–29.56	**−**20.03–7.24	3.04–27.74	**−**11.40–22.39	**−**1.00–38.46	**−**2.30–13.79	**−**12.75–16.29	**−**11.80–17.42	10.22–20.53	**−**3.43–21.04	**−**3.69–9.18	1.07–66.54	**−**3.73–32.59
High	**18.67**	**2.24**	**30.80**	**19.66**	**21.21**	**31.89**	**−2.58**	**19.81**	**24.20**	**21.69**	**2.55**	**22.52**	**17.58**	**27.03**	**17.49**	**7.49**	**8.43**	**25.37**	**18.79**	**9.42**	**42.73**	**19.64**
	14.56–22.79	**−**16.57–21.06	21.67–39.94	9.29–30.03	15.94–26.48	13.55–50.23	**−**17.47–12.30	8.84–30.79	13.49–34.90	10.10–33.29	**−**10.39–15.50	10.65–34.38	1.18–33.98	7.42–46.65	9.44–25.53	**−**6.15–21.13	−5.83–22.70	20.07–30.66	7.22–30.36	3.26–15.59	10.21–75.25	2.07–37.20
Very high	**25.62**	**13.01**	**33.00**	**27.33**	**25.82**	**37.63**	**−1.44**	**24.96**	**28.94**	**27.54**	**6.26**	**27.50**	**20.69**	**33.13**	**21.12**	**17.24**	**17.40**	**26.34**	**23.03**	**15.20**	**43.83**	**26.81**
	21.23–30.00	**−**7.29–33.32	23.36–42.64	16.07–38.58	20.33–31.30	18.41–56.86	−18.30–15.42	12.24–37.68	16.28–41.60	15.34–39.75	**−**6.84–19.36	15.13–39.88	3.78–37.59	13.23–53.02	12.64–29.59	3.01–31.48	1.99–32.81	18.38–34.29	11.07–34.99	90.1–21.39	10.99–76.67	8.81–44.80
*p*-value	<0.001	0.001	<0.001	<0.001	<0.001	<0.001	<0.001	<0.001	<0.001	<0.001	<0.001	<0.001	<0.001	<0.001	<0.001	<0.001	0.006	<0.001	<0.001	<0.001	<0.001	<0.001
Social interactions																						
0 = extreme, 12 = no problems	**2.20**	**2.51**	**1.99**	**1.60**	**1.72**	**2.81**	**2.10**	**1.57**	**2.15**	**1.39**	**1.56**	**2.08**	**2.43**	**2.39**	**2.30**	**1.72**	**1.64**	**2.44**	**2.29**	**1.47**	**1.48**	**1.59**
	1.87–2.53	1.59–3.43	1.68–2.31	0.99–2.21	1.38–2.06	1.65–3.96	1.27–2.93	0.63–2.51	1.40–2.90	0.39–2.39	0.84–2.28	1.32–2.85	1.59–3.27	1.89–2.89	1.94–2.67	0.81–2.63	0.61–2.67	2.00–2.89	1.61–2.97	1.17–1.77	0.66–2.30	0.69–2.48
*p-value*	<0.001	<0.001	<0.001	<0.001	<0.001	<0.001	<0.001	0.001	<0.001	0.007	<0.001	<0.001	<0.001	<0.001	<0.001	<0.001	0.002	<0.001	<0.001	<0.001	<0.001	<0.001

Abbreviations: CI, confidence intervals; Coeff, Coefficients; MHI-5, SF-36 5-item Mental Health Index. Results based on imputed data (for sample size of countries see Appendix 1 and 2). Models adjusted for age, gender, partnership status, employment status, socioeconomic position, lesion characteristics, unavailability of health services and secondary health conditions. *p*-values from likelihood ratio tests.

**TABLE 4:** Social relationships and low risk of mental health disorders in the 22 countries participating in the International Spinal Cord Injury Community Survey (InSCI): adjusted odds ratios and 95% confidence intervals indicating low risk of mental health disorders by social relationships, stratified by country (InSCI 2017–2019).

	Low risk for mental health disorders (MHI-5 scores >56)																					
	Australia	Brazil	China	France	Germany	Greece	Indonesia	Italy	Japan	Lithuania	Malaysia	Morocco	Netherlands	Norway	Poland	Romania	South Africa	South Korea	Spain	Switzerland	Thailand	United States
	OR 95% CI	OR 95% CI	OR 95% CI	OR 95% CI	OR 95% CI	OR 95% CI	OR 95% CI	OR 95% CI	OR 95% CI	OR 95% CI	OR 95% CI	OR 95% CI	OR 95% CI	OR 95% CI	OR 95% CI	OR 95% CI	OR 95% CI	OR 95% CI	OR 95% CI	OR 95% CI	OR 95% CI	OR 95% CI
Partnership status																						
No partner	Ref	Ref	Ref	Ref	Ref	Ref	Ref	Ref	Ref	Ref	Ref	Ref	Ref	Ref	Ref	Ref	Ref	Ref	Ref	Ref	Ref	Ref
Having a partner	**1.22**	**1.09**	**0.98**	**1.27**	**1.35**	**2.49**	**2.22**	**1.05**	**1.32**	**1.18**	**1.06**	**0.59**	**1.47**	**1.15**	**1.04**	**1.04**	**1.60**	**1.57**	**0.71**	**0.94**	**1.34**	**0.68**
	0.94–1.60	0.56–2.12	0.69–1.40	0.76–2.11	1.02–1.78	1.18–5.27	0.95–5.18	0.49–2.25	0.75–2.32	0.36–3.81	0.53–2.10	0.35–1.01	0.66–3.26	0.72–1.85	0.76–1.42	0.46–2.33	0.65–3.95	1.14–2.17	0.43–1.18	0.68–1.30	0.72–2.50	0.24–1.92
*p*-value	0.136	0.739	0.921	0.355	0.035	0.017	0.066	0.910	0.340	0.785	0.869	0.054	0.341	0.559	0.796	0.925	0.309	0.006	0.193	0.701	0.356	0.472
Living situation																						
Living alone	Ref	Ref	Ref	Ref	Ref	Ref	Ref	Ref	Ref	Ref	Ref	Ref	Ref	Ref	Ref	Ref	Ref	Ref	Ref	Ref	Ref	Ref
Living with others	**0.91**	**0.79**	**1.42**	**0.46**	**1.30**	**0.54**	**1.90**	**0.47**	**1.08**	**4.59**	**1.29**	**2.28**	**1.13**	**0.93**	**1.11**	**0.16**	**0.96**	**1.05**	**0.88**	**1.32**	**0.88**	**0.78**
	0.63–1.30	0.20–3.09	0.80–2.55	0.20–1.02	0.87–1.95	0.20–1.41	0.40–8.97	0.16–1.39	0.48–2.46	0.67–31.36	0.30–5.59	0.67–7.72	0.27–4.69	0.45–1.94	0.68–1.82	0.03–0.94	0.21–4.35	0.70–1.57	0.40–1.95	0.81–2.17	0.19–4.12	0.19–3.24
Living in an institution	**0.70**	Omitted	**0.78**	**0.21**	**0.74**	Omitted	**2.05**	**0.13**	**0.42**	Omitted	**0.95**	Omitted	Omitted	**0.14**	**0.89**	Omitted	**0.24**	**0.85**	**1.05**	**1.20**	**1.69**	Omitted
	0.34–1.42		0.31–1.95	0.03–1.34	0.37–1.45		0.34–12.32	0.01–2.63	0.05–3.38		0.13–6.84			0.04–0.54	0.38–2.09		0.04–1.28	0.21–3.49	0.18–6.00	0.50–2.88	0.26–10.90	
*p*-value	0.590	0.740	0.176	0.066	0.189	0.206	0.695	0.215	0.640	0.120	0.870	0.186	0.862	0.016	0.801	0.042	0.031	0.934	0.942	0.541	0.580	0.734
Belongingness																						
0 = not at all	Ref	Ref	Ref	Ref	Ref	Ref	Ref	Ref	Ref	Ref^a^	Ref	Ref	Ref	Ref	Ref	Ref	Ref	Ref	Ref	Ref	Ref	Ref
1	**1.13**	**2.63**	**2.11**	**0.59**	**1.18**	**4.36**	**1.22**	**0.88**	**1.23**		**0.51**	**4.09**	**1.57**	**1.57**	**0.49**	**3.27**	**0.49**	**1.23**	**0.29**	**0.70**	**0.78**	**14.67**
	0.56–2.26	0.54–12.83	1.07–4.17	0.11–3.10	0.50–2.80	0.23–80.94	0.31–4.77	0.11–6.84	0.34–4.52		0.14–1.91	0.59–28.59	0.20–12.43	0.08–30.39	0.19–1.23	0.26–42.01	0.05–5.15	0.57–2.64	0.06–1.43	0.25–2.01	0.14–4.46	0.26–837.5
2	**1.35**	**0.63**	**4.29**	**1.43**	**2.05**	**5.83**	**0.91**	**1.62**	**2.43**	**12.91**	**1.36**	**4.81**	**1.11**	**1.49**	**1.55**	**1.26**	**1.00**	**1.92**	**0.75**	**1.00**	**2.35**	**16.88**
	0.71–2.57	0.16–2.53	2.27–8.10	0.30–6.77	0.88–4.75	0.37–92.21	0.26–3.23	0.24–11.00	0.79–7.47	0.03–58.49	0.42–4.39	0.78–29.72	0.20–6.22	0.08–27.27	0.72–3.33	0.13–12.41	0.16–6.08	0.97–3.83	0.22–2.56	0.39–2.01	0.46–12.09	0.34–834.3
3	**3.04**	**10.68**	**10.51**	**2.70**	**3.56**	**13.53**	**1.78**	**2.70**	**13.85**	**8.61**	**3.35**	**8.47**	**1.91**	**2.07**	**2.60**	**2.27**	**2.60**	**4.79**	**1.73**	**2.00**	**3.57**	**28.07**
	1.59–5.84	2.62–43.60	5.46–20.22	0.58–12.57	1.58–8.03	0.98–186.60	0.50–6.35	0.40–18.16	3.90–49.20	0.02–35.10	1.07–10.50	1.35–52.97	0.32–11.43	0.11–37.92	1.25–5.43	0.25–20.58	0.45–15.03	2.38–9.64	0.54–5.51	0.81–2.58	0.70–18.18	0.60–1324.6
4 = completely	**4.80**	**4.40**	**16.49**	**3.34**	**4.95**	**24.43**	**2.27**	**4.57**	**7.39**	**27.10**	**6.27**	**13.81**	**3.15**	**4.14**	**3.72**	**4.11**	**1.35**	**6.41**	**2.70**	**4.23**	**3.81**	**68.5**
	2.48–9.30	1.42–13.70	7.89–34.45	0.69–16.28	2.18–11.20	1.86–320.58	0.52–9.82	0.62–33.59	2.18–25.07	0.07–109.57	1.79–22.03	2.35–81.05	0.49–20.47	0.22–76.81	1.79–7.74	0.44–38.51	0.24–7.77	2.95–13.90	0.89–8.23	1.66–10.76	0.71–20.48	1.33–3538.2
*p*-value	<0.001	<0.001	<0.001	0.002	<0.001	0.007	0.493	0.111	<0.001	0.222	<0.001	<0.001	0.381	0.026	<0.001	0.191	0.203	<0.001	<0.001	<0.001	0.012	0.083
Relationship satisfaction																						
Very low	Ref	Ref	Ref	Ref	Ref	Ref^a^	Ref	Ref	Ref	Ref^a^	Ref	Ref	Ref	Ref^a^	Ref	Ref	Ref	Ref	Ref	Ref	Ref	Ref
Low	**1.55**	**0.45**	**2.16**	**5.33**	**1.90**		**0.08**	**0.96**	**1.74**		**0.25**	**1.63**	**0.45**		**1.13**	**0.71**	**0.94**	**0.84**	**1.67**	**0.44**	**0.03**	**4.54**
	0.84–2.84	0.05–4.32	0.26–18.22	0.77–36.83	0.72–5.03		0.01–0.98	0.08–11.32	0.31–9.85		0.02–2.60	0.22–12.22	0.03–7.60		0.25–5.18	0.07–7.51	0.11–8.17	0.39–1.83	0.32–8.73	0.16–1.27	0.00–0.29	0.22–94.4
Medium	**2.94**	**0.62**	**4.71**	**4.65**	**4.61**	**1.30**	**0.20**	**1.31**	**3.86**	**0.45**	**0.28**	**2.49**	**1.65**	**4.87**	**2.41**	**0.94**	**1.89**	**2.18**	**1.78**	**1.30**	**0.34**	**15.36**
	1.67–5.17	0.08–4.56	0.59–37.56	0.76–28.31	1.81–11.77	0.35–4.83	0.02–1.74	0.13–13.63	0.73–20.44	0.06–3.41	0.04–2.10	0.41–15.02	0.12–22.10	1.98–11.96	0.60–9.73	0.13–6.57	0.31–11.71	1.08–4.38	0.37–8.49	0.50–3.38	0.13–0.92	0.59–397.3
High	**5.97**	**1.30**	**17.18**	**10.58**	**8.23**	**5.73**	**1.18**	**3.30**	**9.98**	**0.71**	**1.12**	**5.37**	**10.45**	**11.29**	**7.97**	**1.72**	**2.52**	**5.72**	**4.75**	**3.04**	**0.80**	**30.83**
	3.38–10.52	0.19–8.81	2.16–136.76	1.79–62.59	3.41–19.84	1.66–19.80	0.13–10.10	0.33–33.18	1.87–53.42	0.09–5.42	0.16–7.93	0.93–30.87	0.80–135.68	4.61–27.63	1.99–31.88	0.28–10.64	0.44–14.59	2.80–11.68	1.07–21.10	122–7.63	0.31–2.08	1.49–637.8
Very high	**11.44**	**5.51**	**15.09**	**40.75**	**12.89**	**10.94**	**0.93**	**5.48**	**9.76**	**2.08**	**2.18**	**8.65**	**15.17**	**28.59**	**13.16**	**3.07**	**4.78**	**6.05**	**6.48**	**8.75**	Omitted	**39.90**
	6.14–21.31	0.56–53.76	1.79–126.88	4.98–333.34	4.96–33.46	2.40–50.00	0.07–13.22	0.35–86.30	1.32–71.90	0.21–20.92	0.28–16.66	1.44–51.93	0.96–240.16	7.87–103.88	3.06–56.50	0.42–22.23	0.63–36.04	1.95–18.76	1.37–30.59	3.24–23.65		1.55–1026.2
*p*-value	<0.001	0.033	<0.001	0.001	<0.001	<0.001	<0.001	0.108	<0.001	0.357	<0.001	0.001	<0.001	<0.001	<0.001	0.327	0.366	<0.001	0.001	<0.001	0.001	0.017
Social interactions																						
0 = extreme, 12 = no problems	**1.23**	**1.24**	**1.22**	**1.20**	**1.19**	**1.40**	**1.28**	**1.15**	**1.24**	**1.06**	**1.27**	**1.19**	**1.30**	**1.37**	**1.28**	**1.25**	**1.15**	**1.24**	**1.27**	**1.22**	**1.15**	**1.34**
	1.17–1.28	1.11–1.38	1.17–1.28	1.09–1.32	1.13–1.25	1.20–1.64	1.13–1.47	0.98–1.35	1.12–1.37	0.87–1.30	1.13–1.44	1.09–1.30	1.12–1.52	1.24–1.50	1.21–1.36	1.06–1.46	1.00–1.32	1.17–1.31	1.16–1.38	1.16–1.29	1.03–1.28	1.12–1.59
*p*-value	<0.001	<0.001	<0.001	<0.001	<0.001	<0.001	<0.001	0.093	<0.001	0.566	<0.001	<0.001	0.001	<0.001	<0.001	0.006	0.043	<0.001	<0.001	<0.001	0.016	0.001

Abbreviations: CI, confidence intervals; MHI-5, SF-36 5-item mental health index; OR, odds ratios. Results based on imputed data (for sample size of countries see Appendix 1 and 2). Models adjusted for age, gender, partnership status, employment status, socioeconomic position, lesion characteristics, unavailability of health services and secondary health conditions. p-values from likelihood ratio tests.

^a^ Due to low numbers of cases, lowest and 2^nd^ lowest categories combined for analysis.

## Discussion

This study demonstrates pronounced and consistent associations of functional aspects of social relationships with mental health in persons with SCI across different regions of the world. Persons with higher sense of belongingness, higher relationship satisfaction and fewer problems with social interactions reported markedly better general mental health and a lower likelihood for mental health disorders than those with less favorable quality of social relationships, even after adjustment for injury severity, demographic characteristics and secondary health conditions. Notably, previous studies identified a 4- to 5-point difference on the MHI-5 as the minimum clinically important difference [34, 35] and the pronounced differences in mental health according to the quality of social relationships is thus highly relevant from a clinical perspective. In contrast, differences in mental health by partnership status and the living situation were less distinct and inconsistent across countries.

Our findings on the positive association between functional social relationships and mental health are in line with previous evidence from quantitative studies, including general populations and few available findings from populations with disabilities [3–6, 16]. The main-effects model and the stress-buffering model [36] present two complementary models to explain our findings. The *main-effects model* suggests that functional relationships directly affect mental health by enhancing access to various forms of support, such as access to health-relevant information or emotional encouragement, which is beneficial for mental health. In turn, dysfunctional social relationships directly affect mental health by triggering physiological responses with adverse effects on mental health and wellbeing. For example, a recent systematic review comprising 20 studies concluded that social disconnectedness is associated with increased allostatic load [11], which plays an important role in the development of mood disorders, such as depression or anxiety disorders [37]. The *stress-buffering model* provides an additional explanation for the observed associations, whereby functional social relationships are thought to modulate responses to adversity [4, 36, 38]. High relationship satisfaction, feelings of belongingness and non-problematic social interactions may lead to more positive appraisals of stressful situations, resulting in constructive psychological and behavioral responses, such as favorable emotional regulation and active coping styles [10]. These psychological resources may support mental health by buffering strain, while dysfunctional relationships reduce resources to buffer strain from adversity and therefore put people at risk for poorer mental health. Our study supports the assumption that the association between social relationships and mental health is universal across different cultures, since we observed quite consistent findings over all countries. We interpret this finding as a confirmation of fundamental human needs theories, stating that interpersonal attachment and belongingness are universal and basic human needs with relevance to mental health [39].

The quantitative method of our study does, however, only allow detecting associations on a group-level and cannot contribute to disentangle individually different underlying mechanisms linking specific resources provided by social relationships to mental health in our specific sample. The above-mentioned well-established explanations on the health effects of social relationships from general populations should therefore be complemented by qualitative findings on the lived experience of persons with SCI. A meta-synthesis of qualitative findings in persons with SCI revealed for example that social relationships have a particularly important role for re-establishing self-worth after injury, as functional relationships to close persons were relevant resources for recognizing that the injury did not change the essence of their person, that their existence was valuable for others, and that they were still able to contribute to society despite potentially severe physical impairments [40]. It is assumed that social relationships support the adaptation process after SCI and act via the strengthening of self-worth, recognition, and feelings of usefulness on mental health outcomes in persons with SCI.

Our study further shows weak and inconsistent associations between the structural aspects of relationships (i.e., partnership status, living situation) and mental health, a tendency that has been described earlier for populations experiencing physical disabilities [15, 16]. Having a partner or living with others was related to better mental health in some countries and related to lower mental health in others. Interestingly, results were also mixed among countries with cultural similarities or comparable level of economic development. The inconclusiveness of results may point to the fact that crude information on partnership status and living situation is not decisive for mental health, as the quality of the relationship with the partner or with people sharing a household are pivotal for mental health. Earlier findings from populations with SCI showed, for example, that reciprocity in the partnership and partner relationship quality was strongly linked to mental health [15]. Findings from the general population have demonstrated that marital satisfaction relates to mental health, with poor marital satisfaction being detrimental for mental health [41]. Similarly, reasons for living alone (i.e., result of free choice vs. result of constraints), the satisfaction with the living situation and the relationship quality to the people in the household might be more critical for mental health than just the fact of living alone or living with others. Further studies taking into account qualitative aspects of the partner relationship or the living situation might lead to more conclusive results.

### Potential Practical Implications

Based on our findings emphasizing the importance of social relationships for mental health in persons with SCI, interventions targeting functional aspects of social relationships should be considered in rehabilitation programs to reduce long-term mental health problems in persons experiencing physical disabilities. Results from a qualitative study on persons with SCI and their partners highlighted for example that specific education and training are needed to support coping and communication skills to strengthen partner relationships after the onset of SCI to counteract negative effects on relationships caused by overprotective behaviours, asymmetrical dependency, loss of sexuality and intimacy, and difficulties in psychological adaptation [42]. Promising results of interventions to strengthen partner relationships and functional social relationships were reported for workshop-based interventions [43], for kindness- and gratitude-based positive psychology interventions fostering positive social interaction with peers for improving relationship satisfaction [44], and for the reduction of loneliness by strengthening present relationships, enabling individuals to overcome functional impairments to maintain contact with friends and family, or by creating opportunities to make new connections through group-based or peer-to-peer activities [45]. Furthermore, cognitive behavioral therapy was shown effective to enhance perceived social support and mental health after myocardial infarction [46] and is described as promising approach to intervene on maladaptive social cognitions in relation to feelings of loneliness or low belongingness [45]. A recent qualitative study on perceived barriers and facilitators for educational programs in persons with SCI showed that the involvement of peers and families in educational activities and personal motivation and commitment were key for the effectiveness of educational activities [47]. It thus seems essential to provide adequate information on the importance of social relationships for mental health in order to enhance personal commitment and willingness of families and persons with SCI to participate in educational programs.

### Strength and Limitations

This study contributed to current evidence in disability research by investigating structural and infrequently studied functional aspects of social relationship in relation to mental health in different countries across the world. We used state-of-the-art statistical methods and the adjusted analysis were based on DAGs, allowing for drawing causal inference. The measurement instrument to assess general mental health has been validated in different regions of the world and among persons with SCI [28] and the study is based on a large sample.

Various methodological limitations need to be discussed when interpreting our findings. Sampling bias might be an issue because only eight countries applied random sampling and 14 countries relied on convenience sampling. Although this may lead to limited generalizability to the total population of individuals with SCI in participating countries, our study does not focus on the epidemiological description of mental health, but rather on the detection of associations between social relationships and mental health. As we did not collect information on social relationships or mental health in non-responders, we cannot evaluate whether survey participation was independent of those constructs. Results of country-specific analysis need to be interpreted cautiously as small samples in some countries might limit the power to detect meaningful associations. In some countries, effect sizes were large but had very wide confidence intervals, indicating potential power issues. Further, it is not possible to assess whether the self-report nature of data lead to biased responses, since for example, information on mental health may be subject to social desirability bias. Also, it is important to mention that this study only investigated three specific aspects of functional relationships assessed with five items, and we therefore cannot make the claim to be representative for the full spectrum of functional aspects of social relationships. Moreover, reverse causation cannot be excluded as people with mental health problems might experience more difficulties in maintaining good quality relationships.

### Conclusion

Our findings highlight the importance of functional aspects of social relationships for mental health in people experiencing physical disability resulting from an SCI, as feelings of belongingness, satisfaction with relationships and few restrictions in social interactions were identified as important resources for mental health in different geographical settings. Future research to reduce the burden of mental health disorders could explore currently promising interventions to support persons with physical disabilities in establishing and maintaining good quality social relationships.

## Data Availability

The datasets presented in this article are not readily available because the 22 countries participating in this survey hold the rights for data use. Requests for data use of third parties need to be negotiated with respective countries. Requests to access the datasets should be directed to insci@paraplegie.ch.
