# Supplementary material for: Are Social Relationships an Underestimated Resource for Mental Health in Persons Experiencing Physical Disability? Observational Evidence From 22 Countries
Source: Int J Public Health. 2021 Apr 16;66:619823. doi: 10.3389/ijph.2021.619823 (PMC8565297; doi:10.3389/ijph.2021.619823)
Supplement: Supplementary file 1 [file DataSheet1.pdf]

Electronic Supplementary Material

Supplementary Table 1. Structural and functional aspects of social relationship in the participants of the InSCI community survey (n=12,330)

	**Total**	**In partnership**		**Living situation**				**Belongingness**		**Relationship satisfaction**		**Problems with social interactions**	
				Living alone	Living with others	Living in an institution		Range 0-4, higher scores higher belongingness		Range 0-4, higher scores higher satisfaction		Range 0-12, higher score fewer problems	
Country	N	N (%)	*m*	N (%)	N (%)	N (%)	*m*	Mean (SD); Median (IQR)	*m*	Mean (SD); Median (Q1, Q3)	*m*	Mean (SD); Median (Q1, Q3)	*m*
Australia	1,579	931 (59.2)	0.3	361 (23.0)	1,153 (73.5)	55 (3.5)	0.6	2.8 (1.1); 3 (2-4)	3.9	2.6 (1.1); 3 (2-3)	4.2	8.1 (3.2); 9 (6-11)	3.8
Brazil	201	103 (51.2)	0	13 (6.5)	186 (93.5)	0 (0)	1.0	3.0 (1.4); 4 (2-4)	0.5	2.8 (0.8); 3 (2-3)	0	6.7 (3.5); 6 (4-9)	0
China	1,354	1153 (85.1)	0	72 (5.3)	1,245 (92.3)	32 (2.4)	0.4	2.4 (1.1); 2 (2-3)	0	2.6 (0.7); 3 (2-3)	0	7.4 (3.7); 8 (4-11)	0
France	413	231 (56.3)	0.7	128 (31.8)	269 (66.8)	6 (1.5)	2.4	2.9 (1.0); 3 (2-4)	4.6	2.7 (0.9); 3 (2-3)	3.9	8.7 (2.8); 9 (7-11)	4.6
Germany	1,617	981 (62.4)	2.8	348 (22.6)	1,139 (73.9)	54 (3.5)	4.7	2.9 (1.1); 3 (2-4)	6.3	2.8 (1.0); 3 (2-3)	4.1	6.1 (3.4); 6 (3-9)	7.0
Greece	200	93 (46.7)	0.5	44 (22.1)	154 (77.4)	1 (0.50)	0.5	3.1 (1.1); 3 (3-4)	8.0	2.7 (1.0); 3 (2-3)	6.5	9.2 (3.0); 10 (8-12)	2.5
Indonesia	201	138 (69.4)	1.0	10 (5.1)	156 (78.8)	32 (16.2)	1.5	2.3 (1.2); 2 (2-3)	2.0	2.5 (0.8); 3 (2-3)	2.5	8.0 (3.5); 8 (6-11)	1.5
Italy	206	105 (51.0)	0	35 (17.1)	166 (81.0)	4 (2.0)	0.5	2.5 (1.2); 3 (2-3)	6.3	2.5 (1.0); 3 (2-3)	5.8	7.6 (3.1); 8 (5-10)	4.4
Japan	302	179 (60.5)	2.0	47 (15.7)	248 (82.7)	5 (1.7)	0.7	2.4 (1.2); 2 (2-3)	2.0	2.3 (0.9); 3 (2-3)	1.0	8.1 (3.2); 8 (6-11)	3.6
Lithuania	218	132 (60.6)	0	27 (12.4)	190 (87.6)	0 (0)	0.5	3.3 (0.8); 3 (3-4)	0.9	2.7 (1.0); 3 (2-3)	1.4	8.3 (2.5); 9 (7-10)	0.5
Malaysia	298	141 (47.5)	0.3	15 (5.2)	260 (90.0)	14 (4.8)	3.0	2.6 (1.2); 3 (2-4)	3.7	3.0 (0.9); 3 (3-4)	4.0	8.6 (2.8); 9 (7-11)	3.7
Morocco	385	165 (42.9)	0	17 (4.4)	365 (94.8)	3 (0.8)	0	3.0 (1.2); 4 (2-4)	0	2.8 (1.0); 3 (2-3)	0	7.1 (3.0); 8 (5-9)	0
Netherlands	260	179 (69.4)	0.8	59 (23.1)	191 (74.8)	5 (2.0)	1.9	2.8 (1.1); 3 (2-4)	4.2	3.0 (0.9); 3 (3-4)	3.9	8.9 (2.9); 10 (7-12)	5.0
Norway	610	394 (65.1)	0.8	174 (28.9)	413 (68.6)	15 (2.5)	1.3	3.2 (0.9); 3 (3-4)	2.5	2.7 (0.8); 3 (2-3)	2.1	8.9 (2.8); 9 (7-12)	1.6
Poland	971	508 (52.6)	0.5	119 (12.4)	806 (83.7)	38 (4.0)	0.8	3.1 (1.1); 3 (2-4)	2.8	2.7 (0.9); 3 (2-3)	2.8	8.3 (3.2); 9 (6-11)	2.3
Romania	216	89 (41.2)	0	23 (10.6)	192 (88.9)	1 (0.5)	0	3.1 (1.1); 3 (2-4)	1.9	2.9 (0.9); 3 (3-3)	1.4	7.8 (3.0); 8 (5-10)	1.9
South Africa	200	44 (22.1)	0.5	13 (6.5)	132 (66.0)	55 (27.5)	0	3.0 (1.1); 3 (2-4)	1.5	2.7 (0.9); 3 (2-3)	1.0	9.0 (2.9); 10 (7-12)	0
South Korea	890	432 (49.0)	1.0	249 (28.6)	613 (70.4)	9 (1.0)	2.1	2.3 (1.1); 2 (2-3)	2.1	2.1 (0.9); 3 (2-3)	4.0	6.0 (3.3); 6 (4-8)	1.4
Spain	417	242 (58.0)	0	65 (15.7)	341 (82.4)	8 (1.9)	0.7	3.2 (1.1); 4 (3-4)	2.2	2.9 (1.0); 3 (2-4)	1.2	8.6 (3.2); 9 (6-12)	2.4
Switzerland	1,269	749 (59.2)	0.3	359 (28.9)	840 (67.6)	44 (3.5)	2.1	3.1 (1.0); 3 (3-4)	1.2	3.0 (0.9); 3 (3-4)	0.7	7.8 (3.3); 8 (5-11)	6.2
Thailand	320	141 (44.1)	0	13 (4.1)	284 (88.8)	23 (7.2)	0	2.6 (1.0); 3 (2-3)	1.3	2.8 (0.7); 3 (2-3)	0.6	9.7 (2.5); 11 (8-12)	0.9
United States	203	106 (52.5)	0.5	39 (19.2)	163 (80.3)	1 (0.5)	0	2.9 (1.0); 3 (2-4)	2.0	2.8 (1.0); 3 (2-4)	1.5	9.2 (2.8); 10 (7-12)	1.5

Abbreviations: IQR: interquartile range; m: missing values in % of countries total population; SD: Standard deviation. Descriptive values based on crude data.

Supplementary Table 2. Mental health in participants of the InSCI community survey (n=12,330)

	Total		General mental health (MHI-5 scores 0-100)	Low risk of mental health disorder (MHI-5 scores >56)
**Country**	N	*m*	Mean (SD); Median (IQR)	N (%)
Australia	1,579	3.5	68.1 (21.4); 72 (56-86)	1,072 (70.3)
Brazil	201	0	62.0 (22.5); 62 (46-80)	120 (59.7)
China	1,354	0	63.6 (18.7); 68 (50-78)	864 (63.8)
France	413	3.9	67.6 (18.9); 70 (56-82)	291 (73.3)
Germany	1,617	12.6	66.4 (21.2); 72 (54-84)	967 (68.4)
Greece	200	2.0	63.1 (25.2); 68 (50-84)	122 (62.2)
Indonesia	201	1.0	67.2 (18.2); 68 (56-80)	135 (67.8)
Italy	206	1.9	60.9 (19.2); 62 (50.76)	110 (54.5)
Japan	302	2.0	63.4 (20.3); 68 (50-78)	180 (60.8)
Lithuania	218	1.4	71.7 (19.0); 80 (66-84)	172 (80.0)
Malaysia	298	4.4	68.4 (17.2); 70 (56-82)	207 (72.6)
Morocco	385	0	57.3 (23.0); 56 (40-76)	188 (48.8)
Netherlands	260	5.0	74.0 (19.4); 78 (62-88)	200 (81.0)
Norway	610	3.0	73.2 (17.7); 78 (62-88)	474 (80.1)
Poland	971	2.0	60.8 (19.6); 62 (50-76)	560 (58.8)
Romania	216	1.4	69.6 (19.3); 74 (62-86)	161 (75.6)
South Africa	200	0.5	63.9 (21.1); 66 (50-80)	127 (63.8)
South Korea	890	1.9	58.1 (21.5); 56 (44-74)	432 (49.5)
Spain	417	4.1	66.1 (23.8); 72 (50-85)	267 (66.8)
Switzerland	1,269	5.0	73.3 (17.7); 78 (62-88)	962 (79.8)
Thailand	320	1.6	68.2 (19.0); 72 (56-82)	222 (70.5)
United States	203	2.5	73.1 (18.5); 76 (62-88)	164 (82.8)

Abbreviations: IQR: interquartile range; m: missing values in % of countries total population; MHI-5: 5-item Mental Health Index from SF-36, higher scores indicate better mental health; SD: Standard deviation.
Descriptive values based on crude data.
